# A Brief Introduction to Some Aspects of the Fluid–Mosaic Model of Cell Membrane Structure and Its Importance in Membrane Lipid Replacement

**DOI:** 10.3390/membranes11120947

**Published:** 2021-11-29

**Authors:** Garth L. Nicolson, Gonzalo Ferreira de Mattos

**Affiliations:** 1 Department of Molecular Pathology, The Institute for Molecular Medicine, Huntington Beach, CA 92647, USA; 2 Laboratory of Ion Channels, Biological Membranes and Cell Signaling, Department of Biophysics, Facultad de Medicina, Universidad de la República, Montevideo 11600, Uruguay; ferreira@fmed.edu.uy

**Keywords:** lipid interactions, membrane domains, extracellular matrix, lipid rafts, membrane fusion, membrane structure, cytoskeletal interactions, membrane vesicles, endosomes, membrane dynamics

## Abstract

Early cell membrane models placed most proteins external to lipid bilayers in trimolecular structures or as modular lipoprotein units. These thermodynamically untenable structures did not allow lipid lateral movements independent of membrane proteins. The Fluid–Mosaic Membrane Model accounted for these and other properties, such as membrane asymmetry, variable lateral mobilities of membrane components and their associations with dynamic complexes. Integral membrane proteins can transform into globular structures that are intercalated to various degrees into a heterogeneous lipid bilayer matrix. This simplified version of cell membrane structure was never proposed as the ultimate biomembrane description, but it provided a basic nanometer scale framework for membrane organization. Subsequently, the structures associated with membranes were considered, including peripheral membrane proteins, and cytoskeletal and extracellular matrix components that restricted lateral mobility. In addition, lipid–lipid and lipid–protein membrane domains, essential for cellular signaling, were proposed and eventually discovered. The presence of specialized membrane domains significantly reduced the extent of the fluid lipid matrix, so membranes have become more mosaic with some fluid areas over time. However, the fluid regions of membranes are very important in lipid transport and exchange. Various lipid globules, droplets, vesicles and other membranes can fuse to incorporate new lipids or expel damaged lipids from membranes, or they can be internalized in endosomes that eventually fuse with other internal vesicles and membranes. They can also be externalized in a reverse process and released as extracellular vesicles and exosomes. In this Special Issue, the use of membrane phospholipids to modify cellular membranes in order to modulate clinically relevant host properties is considered.

## 1. Introduction: Cell Membranes

Cell or plasma membranes are the first cellular barriers encountered by extracellular ions, molecules, lipid vesicles and globules, viruses and other cells [1]. Cell membrane interactions with extracellular molecules determine how individual cells process nutrients, initiate cellular signaling and respond to and maintain normal cellular physiology [1,2]. Thus, cell membranes are important filters that provide a cellular barrier and continuity, while selectively transmitting signals, nutrients and substances from outside to inside cells and then to various cellular organelles. In addition, cells release signals and molecules to adjacent cells, tissues and distant organs, including lipid vesicles and globules containing other molecules (proteins, DNA etc.), and in doing so, they can condition host micro- and macro-environments [3,4]. Cells are also compartmentalized into organelles by various complex intracellular membranes that are also responsible for the biosynthesis of various molecules, energy production, replication, transportation, reutilization, destruction, secretion and other activities that are essential in cell and tissue organization and maintenance [3,4].

A basic concept in the organization of cellular membranes is that they are made up of amphipathic molecular components that associate into macro-structures that exclude water interactions on their hydrophobic surfaces. In contrast, the hydrophilic portions of their structures interact with the aqueous environment and other hydrophilic molecules [5,6]. This concept was implied by the experiments of Langmuir, who used oil layers on aqueous surfaces and measured surface tensions [5]. In 1925, Gorter and Grendel [7] used Langmuir’s methods to assess the notion that that red blood cells were surrounded by two layers of membrane lipids, which was consistent with Fricke’s estimate from cell membrane capacitance experiments that cell membranes were approximately 4 nm thick [8]. Edidin has discussed the historical concepts that cell membranes are composed of phospholipid bilayers plus some membrane proteins [9]. Using this same concept, Danielli and Davson [10] proposed that cellular membranes were lipid bilayers, as proposed by Gorter and Grendel [7], that interacted with flattened or beta-sheet proteins via the hydrophilic head groups of membrane phospholipids. Using primarily electron microscopy of erythrocytes and other cells fixed and stained with excess heavy metals, such as osmium tetroxide, Robertson visualized a trimolecular structure (protein–lipid–protein) that was named the “Unit Membrane” [11]. In contrast, a repeating subunit model of lipoproteins was proposed by Benson that did not have a matrix bilayer of phospholipids [12].

The concept that the matrix of cellular membranes contains amphipathic phospholipids that self-assemble to form lipid bilayers due to the energy provided by the hydrophobic effect and van der Waals forces has evolved over the years [3,6,13]. Integral membrane proteins assemble into this matrix and interact with membrane lipids through hydrophobic forces and much less so through hydrophilic forces between lipid head groups and hydrophilic amino acids of membrane proteins [3,6,9,13,14]. The state of membrane phospholipids is important in this process, because the membrane insertion of proteins appears to be, at least in some cases, limited to regions of membranes where a fluid lipid matrix allows protein– or protein complex–lipid hydrophobic interactions, molecular sorting and, eventually, lateral movements of membrane components [13,14,15].

**Figure 1:**
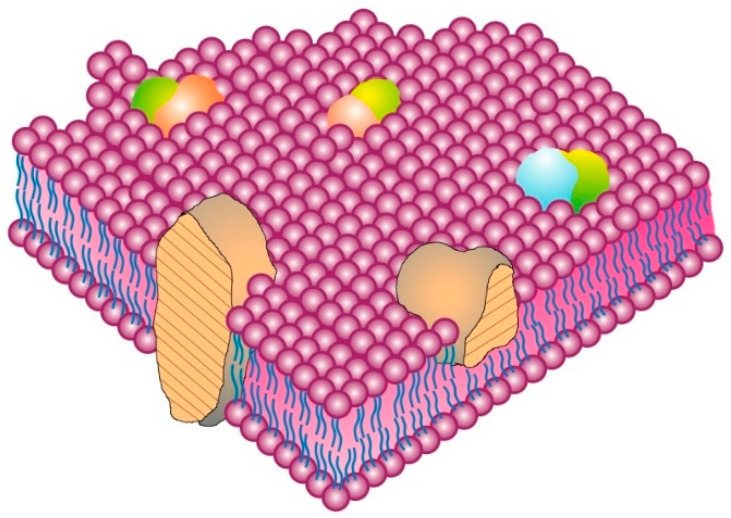
The Singer–Nicolson Fluid–Mosaic Membrane Model of cell membrane structure as proposed in 1972. In this view of a cell membrane, the solid bodies with stippled cut surfaces represent globular integral membrane proteins randomly distributed in the plane of the membrane. Some integral membrane proteins form specific integral protein complexes as shown in the figure. Integral proteins are represented in a fluid lipid bilayer. The model does not contain other membrane-associated structures or membrane domains (Modified from Singer and Nicolson [6]).

There are several different types of membrane proteins, but they can basically be assigned to three classes: integral, peripheral [6,13] and, introduced later, membrane-associated proteins [15]. The classic integral or intrinsic membrane proteins in the 1972 Singer–Nicolson model [6] were shown as globular in structure and bound to membranes by mainly hydrophobic forces (Figure 1). The integral membrane proteins were thus intercalated into the membrane lipid bilayer and not attached to it as in previous membrane models [10,11]. In contrast, peripheral membrane proteins were proposed to be attached to membranes mainly by electrostatic and other forces [6]. Peripheral membrane proteins were proposed as removeable from membranes without destroying basic membrane nanostructure and continuity, and they were thought to serve as important components in providing membrane stability; curvature; scaffolding; tethering; and other characteristics, such as attachment points for enzymes and signaling complexes [15,16,17]. Later, membrane-associated proteins were added that were not generally associated with the hydrophobic matrix of membranes. Their transient interactions with membranes occurred through protein or lipid attachments instead of intercalation into a membrane lipid matrix, and their function was mainly to provide connections with other cellular components, such as enzymes, protein complexes, cytoskeletal elements and other components and structural integrity [3,15].

Cell membranes can be disturbed, distorted, deformed, compressed or expanded by different forces, and diverse molecules can cause these physical perturbations [14,15,16,17]. For example, certain peripheral membrane proteins can bind to and cause the deformation of membranes by forming crescent-shaped, helical bundles that bind to membranes via electrostatic and some hydrophobic forces, causing membrane curvature as a result of flexing and bending membranes to fit these peripheral protein structures [16,17]. In contrast, membrane-associated proteins, for the most part, act indirectly on membranes, usually through intermediate protein or lipid attachments. Although some membrane-associated proteins can be isolated with and loosely attached to cell membranes, they are not truly membrane proteins due to their transient interactions with membranes and their irrelevance to basic membrane nano-scale structure [3,15]. These membrane-associated proteins can include cytoskeletal and signaling structures at the inner cell membrane surface, or at the outer surface, they can include certain extracellular matrix and stromal components. Some cytoplasmic membrane-associated components are quite dynamic and can stabilize or destabilize cellular membranes and connect to other intracellular structures and prevent membrane components from undergoing rapid lateral movements. Alternatively, they can also be involved in translocating membrane complexes via energy-dependent contracting movements, events that can eventually lead to cell polarity, endocytosis or other cellular processes. Membrane-associated proteins are especially important in maintaining certain cellular activities, such as cell adhesion and motility, growth, endocytosis and other important cellular actions [3,4,15,18,19,20,21,22].

## 2. Fluid–Mosaic Model of Membrane Structure

The most accepted rudimentary or nanometer scale model of cell membrane structure, the Fluid–Mosaic Membrane Model, was first proposed in 1972 (Figure 1) [6]. Although this is an oversimplified model that was never intended to explain all aspects of membrane structure and dynamics, it was useful in describing some of the important elements of nano-scale cell membrane architecture, continuity, cooperativity and asymmetry [6,9,13,14,15,16,17,18,19,20,21,22,23,24,25]. The essential elements of the Fluid–Mosaic Membrane Model have proven to be remarkably consistent with experimental results on the fundamental properties of biological membranes, but it was inevitable that the original model could not explain all of the properties of membrane structure and dynamics found in various cellular membranes [18,19,20,21,22,23,24,25,26,27,28]. For example, the concept that membrane mosaic structures or membrane domains, such as lipid rafts, as well as cell membrane-associated structures, such as actin-containing filaments, microtubules and other structures, are important in controlling membrane properties and directing the dynamics of certain cell membrane components was ascertained years after the Fluid–Mosaic Membrane Model was first presented [20,21,22,23,24,25,26,27]. This has resulted in completely contrary suggestions that several membrane models are necessary to explain basic membrane structure and dynamics [24] or that there is no membrane model that can explain cell membrane structure and dynamics at the nano-scale level [25]. We do not share those opinions.

**Figure 2:**
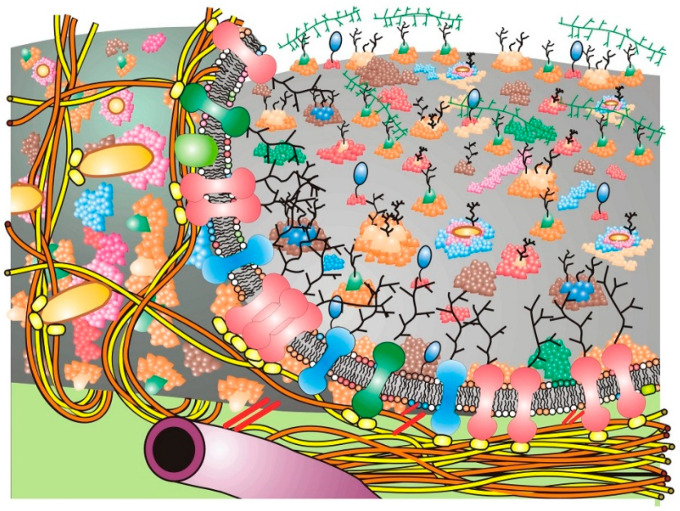
The figure represents a cell or plasma membrane that contains membrane domain structures and membrane-associated cytoskeletal and extracellular structures. The cell membrane has been pealed back at the left to reveal the bottom cell membrane surface and membrane-associated cytoskeletal elements that form barriers (corrals) that limit the lateral motions of some of the integral membrane proteins. In addition, membrane-associated cytoskeletal structures are shown indirectly interacting with integral membrane proteins at the inner membrane surface along with matrix or extracellular matrix components at the outer surface. Although this diagram presents possible schemes of integral membrane protein mobility restraint, it does not accurately represent the sizes and structures of integral membrane proteins, lipid domains or membrane-associated cytoskeletal structures (modified from Nicolson [23]).

With time, updates of the Fluid–Mosaic Membrane Model have made the basic representation of membrane structure far more complex, compact and much less homogeneous looking than the original scheme (cf. [6] with [9,15,20,21,22,23]). The newer proposals on general cell membrane structure contain additional information, such as proposals on membrane asymmetry, protein and lipid associations, membrane complexes and their dynamic segregation into various membrane domains. They can also include trans-membrane signaling complexes, cytoskeletal and stromal interactions and induced dynamic changes in membrane organization, along with other additions. These, among other changes, have now made newer cell membrane schemes much more complex and compact than the original Fluid–Mosaic Membrane Model (for example, Figure 2) [15,19,20,21,22,23,24,25,26]. Importantly, the arrangements of many membrane lipids and proteins into less freely mobile structures, such as lipid–lipid and lipid–protein membrane domains, have maximized the mosaic nature of cell membranes with less fluid areas of freely mobile membrane lipids and proteins than presented in the original Singer–Nicolson model [23,27,28,29,30]. However, the basic nano-scale organization first presented in the Fluid–Mosaic Membrane Model has generally survived, albeit in different overall organizational schemes, and the current models are certainly more crowded and complex than the original proposal [23,27,29,30]. To add to this complexity, Kusumi’s [20,21] concept of a dynamic hierarchical cell membrane organization has made an already complicated description of cell membrane organization even more complex, but necessary, in order to explain newer data on the types of mobility and restrictions of mobility of membrane components and their assembly into ever more complex structures.

The spontaneous, dynamic sorting of membrane components into membrane domains of specific compositions and mobilities was thought to be initially based primarily on hydrophobic and some hydrophilic interactions [15]. Such dynamic sorting avoids hydrophobic mismatches between lipids and proteins, thus preventing unsustainable membrane distortions or areas of membrane weakness [31]. The original Fluid–Mosaic Membrane Model also accounted for cell membrane asymmetry [3,16]. Every cell membrane studied thus far has been found to be asymmetric in terms of the display of membrane components on the interior and exterior sides of membranes [3,9,15,16,17,20,21,22,23,24,25].

An important aspect of cell membrane structure that was first hypothesized, albeit with limited evidence, in the original Fluid–Mosaic Membrane Model was the presence of oligomeric protein/glycoprotein structures in the membrane (see Figure 1 of [6] or Figures 1 and 2 of [15]). Some early evidence was the finding of different antigen distributions—dispersed [26] or micro-clustered [27]—on the same cell type. That initial notion has now become completely refined based on new evidence using state-of-the-art technology for studying the localization and dynamics of single molecules on cell surfaces at the nanometer level [28,29]. An important concept by Garcia-Parajo and colleagues envisions that many if not most cell membrane proteins and glycoproteins exist dynamically (on average) in small nanostructures or nanoclusters in the membrane [28]. Ma et al. have developed a method (Förster Resonance Energy Transfer sensing) that, when combined with single-particle tracking by fluorescence microscopy, can detect the intermolecular associations of neighboring proteins and their clustering events at high spatial and temporal resolutions [29]. Using this method, they were able to map the individual movements of proteins and their clustering events on live T cells and found that many receptors were already present in dense ‘nanoclusters’, and these clusters of receptors had the highest signaling efficiencies [30]. Membrane protein clustering into specific domains also appears to involve interactions with lipid domains, electrostatic interactions between proteins and lipids, inner membrane surface protein scaffolding and other properties of membrane constituents (reviewed in [31]).

## 3. Membrane-Associated Cytoskeletal and Extracellular Matrix Interactions

The original 1972 Fluid–Mosaic Membrane Model did not show important membrane interactions or associations with intracellular or extracellular elements [6], although subsequent modifications of this model included these types of interactions [15,20,21,22,23,24,25]. Cytoskeletal and extracellular matrix interactions are known to alter cell membrane macrostructure by restricting the dynamics or lateral movements of membrane proteins and glycoproteins, and cytoskeleton linkages are also involved in energy-dependent movements of attached membrane components and platforms [3,15,20,21,22,23,24,25,30,31,32,33,34]. Membrane linkages to cytoskeletal elements are also essential in maintaining cell polarity and overall membrane organization and dynamics [3,23,30,31,32,33,34]. Under certain circumstances, they can drive specific cellular properties, such as cell adhesion, movement, endocytosis, exocytosis and many other properties [3,23,28,29,30,31,32,33,34].

Cell membrane receptor clustering, domain formation, submembrane plaque assembly, membrane distortion and internalization, the acidification of the resulting endosomes, the degradation of endosome contents and the recycling of membrane components are all parts of the normal membrane salvaging process [3,23]. An important concept in more recent versions of the Fluid–Mosaic Membrane Model is that the distribution and mobility of integral membrane components can be impaired or selectively anchored by intracellular components or cell–cell, extracellular matrix and stromal interactions, resulting in cell membrane heterogeneity and cell polarity [3,9,15,20,21,22,23,24,25,32,33,34]. Such restraint of the mobility and dynamics of membrane components is thought to be an important element of membrane and cell physiology [21,22,23,24,25,32,33,34,35,36,37,38].

Cells communicate through membrane receptor complexes that can be immobilized by various interactions at the cell surface. Some communication signals are transmitted through dynamically assembled membrane-associated networks that transiently include the cytoskeleton. As mentioned above, the cytoskeleton can also generate mechanical forces that can move membrane components, membrane platforms and even entire cells or inhibit their movements, and they can help cells resist exterior mechanical forces [21,22,23,35,36,37,38,39,40,41]. The serial assembly of specialized cellular elements in and around membranes (integral membrane proteins and lipids, peripheral membrane proteins, adaptor proteins, cytoskeletal elements etc.) may be essential in the conversion of biochemical signals into the mechanical forces that are important in cellular behavior, tissue maintenance and cell movement [36,37,38,39,40]. Although membrane peripheral proteins have been identified as components involved in cell membrane–cytoskeletal interactions [20,21,22,23,37,38,39], membrane lipids are also important in these interactions, as well as in the formation of specialized lipid signaling domains called ‘lipid rafts’ [35,40,42,43,44,45]. Lipid rafts or, at least, specialized lipid domains were hypothesized years before actual experimental evidence for their existence was obtained (cf. [15] with [39,40,41,42,43,44,45]).

Specialized cell membrane domains have been proposed to be dynamic structures that can be generated by the specific interactions of bulk membrane components, extracellular ligand or ion binding, cytoskeletal interactions and other events that can result in the assembly of complex transmembrane structures or platforms [32,33,34,35,36,37]. An example of this is the formation of glycosylphosphatidylinositol (GPI) anchors at the cell surface in the domains or rafts that tether specific GPI-bound proteins [46,47,48,49,50]. These GPI-anchored proteins can exist in different forms, depending on the context and tissue in which they are expressed, and they are known to be involved in various cellular processes, such as cell signaling and adhesion [47,49]. Interestingly, individual GPI-anchored proteins display different modes of lateral movements: essentially stationary, free diffusion, anomalous diffusion and transiently confined diffusion [48]. This is discussed further in Section 5. At the inner membrane surface, some GPI lipid domains appear to be dynamically linked to cortical actin-containing cytoskeletal structures, which may explain some of the GPI domain organization and mobilities and their spatiotemporal regulation [49,50]. How such nanoclusters could be involved in events such as cell spreading and possibly cell movement has been examined by Kalappurakal et al. [51]. These authors examined the role of GPI-anchored protein nanoclustering in cell spreading. They found that a membrane receptor signaling pathway directs membrane protein nanocluster formation. This occurs by the binding of Arg-Gly-Asp motif-containing ligands to the cell surface β1-integrin receptor, which eventually activates src and focal adhesion kinases, resulting in RhoA signaling. This cascade triggers actin nucleation via specific formins that, along with myosin activity, drive the nanoclustering of membrane proteins with actin-binding domains. Eventually, this cascade results in the coupling of the cell’s actomyosin machinery to inner leaflet cell membrane lipids, generating functional GPI-anchored protein nanoclusters that are involved in cell spreading [49,51].

Macromolecular membrane complexes on the cell surface can also recruit specific peripheral and membrane-associated proteins at the inner cell membrane surface to form transmembrane domains, platforms or plaques that are competent for initiating cellular signaling via structural or enzymatic processes or undergoing further attachments to cytoskeletal elements [47,48,49,50,51]. This can result in membrane reorganization, immobilization, signaling events or internalization in endosomes [21,22,23,24,47,48,49,50,51]. We have to consider that cell membranes are essentially completely integrated mechano-structures that exist within single cells, groups of cells and tissues [3,23,33,34,35,36]. Cell membranes are continuously interacting with and linking to various structural components inside and outside cells while receiving signals and contact information from the microenvironment, and they filter and pass these signals on to stimulate appropriate cellular responses. Cell membranes also send messages into the extracellular environment, maintaining cell polarity, cell mechanical properties and tissue barriers while undergoing constant turnover and reassembly of their components.

Over time, the basic nano-scale organization of cell membrane models has evolved significantly from the original models of a rather homogeneous-looking structure (Figure 1) [6] into models that are dynamic yet contain mosaic structures that comprise specific dynamic domains of varying sizes, compositions and mobilities and that can transform into specific membrane regulatory and mechanical structures or platforms that are then linked to various intra- and extra-cellular components [23,24,25,34]. In Figure 2, a rather simplified schematic of these additions to the Fluid–Mosaic Model is depicted, but one should not take such schemes too seriously, because they shall surely change again over time as more information comes to light.

## 4. Membrane Component Interactions in Cell Membranes

One of the more obvious properties of cell membranes that was not adequately portrayed in the original Fluid–Mosaic Model is that membrane constituents are, in general, nonrandomly distributed. For example, membrane lipids are now accepted as being asymmetrically dispersed on the inner and outer leaflets of plasma membranes and also unevenly distributed in the membrane plane [3,15,16,17,23,25,31,52,53,54]. In addition to membrane phospholipids, non-phospholipid membrane lipid molecules are also distributed nonrandomly. For example, cholesterol can affect membrane lipid distributions, and cholesterol is often found enriched in specific membrane domains [53,54,55]. Cholesterol distribution is thought to be due, in part, to its affinity for both the fluid and solid phases of membrane lipids [42,43,44,45]. Cholesterol partitions into liquid-ordered and disordered phases to roughly the same extent, but this partitioning can differently modify the properties of these dissimilar membrane lipid phases [54,55]. Lipids can also modify certain physical properties of membranes [56]. For example, ceramides and lysophospholipids are known to induce membrane curvature, while other lipids, such as cholesterol, can modify membrane lateral elasticity [52,54,55].

Specific membrane lipids, for example, sphingolipids, are important in the formation of ordered membrane lipid mosaic domains or ‘lipid rafts’ [42,43,44,45]. Along with phosphatidylcholine, sphingomyelins constitute more than one-half of the plasma membrane phospholipids and form the most important partners for cholesterol in lipid rafts and other specialized lipid domains. Such small, ordered membrane raft domains that are formed by the preferential associations of cholesterol and saturated lipids are generally surrounded by liquid-phase lipids, and they are thus capable of membrane lateral mobility [42,43,44,45]. They can also selectively recruit other lipids and proteins into their structures [40,54,56]. The lipids within such lipid mosaic domains are not fixed in place—they are still dynamic and can slowly exchange with bulk membrane lipids, as well as with lipids in other membrane domains. In terms of overall size, lipid domains, such as lipid rafts, are usually less than 300 nm in lateral diameter, and most are mostly within 10–200 nm in diameter [57,58,59,60], but some can be larger, and they can undergo clustering induced by protein–protein and protein–lipid interactions into micrometer sized (>300 nm in diameter) domains [39,40,60].

As mentioned above, lipid domains can also contain some integral and peripheral membrane proteins, and these mixed domains can also change in composition with time. The proteins and glycoproteins that are sequestered into membrane domains or rafts can turn these membrane domains into functional signal transduction platforms that are coupled across the membrane and can initiate immune signaling, host–pathogen interactions, endocytosis, cell death regulation and many other events [35,44,60]. In addition, membrane proteins can have profound effects on membrane lipids. They can locally deform membranes and cause the reorganization of membrane lipids to form new membrane domains, as well as regulate membrane properties, such as charge density and diffusion rates [61].

When integral membrane proteins interact with membrane lipids within cellular membranes, portions of their structures must directly interact with the acyl chains of membrane phospholipids or the hydrophobic portions of these and other membrane lipids. This is accomplished by the concept of hydrophobic matching between different classes of membrane molecules [52,53,56]. The concept of hydrophobic matching between the hydrophobic core of the lipid bilayer and hydrophobic stretches of amino acids in integral membrane proteins is essential for the formation of stable membrane structures. If the hydrophobic portions of their structures are mismatched, an elastic distortion of the lipid matrix around the integral membrane protein can occur [52,53]. This can induce protein conformational changes that can affect protein function and protein–protein interactions. Such membrane proteins can also aggregate to cause super-domains to form in membranes. To add to this, there are other physical forces, such as lateral pressure forces, lateral phase changes, membrane curvature and ionic interactions, among other forces, that are important in determining membrane structure [62,63].

## 5. Restrictions on Membrane Mobility and Membrane Domains

There is ample evidence for various restrictions in the rotational and lateral mobilities of certain membrane components and their residence in or compartmentalization into membrane domains in which there are changes in local compositions, lateral organization and dynamics that are different from the average membrane organization [23,40,45,64]. For example, the lateral movements of integral membrane proteins in the membrane plane are often restricted by multiple cis- and trans-membrane interactions that constrain movements within specific membrane domains [20,21,22,23,24,25]. These include extracellular interactions, such as binding to the extracellular matrix and stroma; the formation of specialized membrane domains (lipid rafts and larger, more heterogeneous domains); and integral protein–glycoprotein complexes. At the inner membrane surface, peripheral membrane barriers, curvature-causing peripheral membrane proteins, cytoskeletal interactions and other obstacles can also control membrane component movements [15,20,21,22,23,24,25,28,42,43,44,45,46,47,48,49]. To accommodate the many diverse structures and interactions that can occur at the inner and outer cell membrane surfaces, cell membranes must contain a number of membrane domains, barriers, platforms and membrane-associated structures [15,20,21,22,23,24,25,39,40,41,42,43,44,45,63,64,65].

With time, all of the current basic models of the cell membrane structure evolved to be more complex than the original Fluid–Mosaic Model, and they also contain additional membrane-associated structures and domains that were impossible to contemplate at the time of the original Fluid–Mosaic proposal [6]. Since these structures were discovered well after the original model was proposed, they are missing in the original Fluid–Mosaic Model [6]. The one aspect that all of the current membrane models have in common is that they are remarkedly more complex than the original Fluid–Mosaic Model, but they still retain some of the basic elements of the original model [20,21,22,23,24,25,28,31,34,40,62].

What is important in current membrane model proposals is that the restraint of mobility of integral glycoproteins or glycoprotein receptors in the cell membrane plane and their presence in specific membrane domains has functional physiological consequences. This concept has attracted an immense amount of attention in the last two decades [20,21,22,23,24,25,37,40,42,43,44,45,60,61,62,63,64]. The lateral movements of some specific membrane proteins and cell surface receptors have been examined, and their movements (or restraint of movements) have been organized into various categories. Some examples of these categories are the following: (*a*) random movement or free diffusion in the fluid portions of the membrane; (*b*) transient movements confined by membrane obstacles made up of protein clusters that have been likened to ‘fence posts’ or ‘pickets’; (*c*) transient movements that are constrained by structural domains or ‘corrals’ circumscribed by cytoskeletal elements and their attachment molecules; and (*d*) directed movements due to attachment to and contraction of the cytoskeleton [20,21,22,23,24]. This has led to descriptions of the various two-dimensional motions of membrane components within and between various membrane domains as (*i*) free Brownian diffusion; (*ii*) anomalous diffusion caused by changes in the lipid nano-environment; (*iii*) channeled diffusion defined by membrane-associated cytoskeletal structures; (*iv*) confined diffusion limited by defined structural ‘corrals’; and (*v*) hop diffusion, where diffusion occurs intermittently and differently between dissimilar domains [20,21,22,64]. Thus, the original description of integral membrane proteins freely diffusing in the membrane plane without regard to different membrane domains or obstructions is limited to only one of these categories [6]. Moreover, the original Fluid–Mosaic proposal of cell membranes did not adequately describe the multiple ways that membrane components can aggregate, separate, move or be restrained from movement in various domains in the plane of the membrane, nor did it describe the types of molecular interactions that can control membrane dynamics [20,21,22,23,24,25,39,40,49,55,64].

Our current concept of cell or plasma membrane dynamics is that substantial portions of integral membrane proteins are in mosaic structures that are incapable of free lateral diffusion in the cell membrane plane, or they may only be transiently available to undergo free movements in the membrane plane [20,21,22,23,24,34,36,47,48,64]. Many cell membrane components are thought to be confined, at least part of the time, to membrane domains circumscribed by barriers within the membrane matrix or barriers attached to membranes, such as cytoskeleton networks, or at the outer surface by interactions with the extracellular matrix or stroma [20,21,22,23,24,25,34,35,36,40,49,55,60,64]. Since cell membranes are dynamic structures, over time, some integral proteins and, separately, some lipids can escape from one or more of these domains and move to adjacent domains. Alternatively, they can escape membrane domains altogether, or they can undergo associations in the membrane plane and become super-sized mosaic structures, preventing extra-domain movements [20,21,22,23,24,25,60,64]. These latter super-sized structures may also be precursors of endosomes brought into cells by endocytosis mechanisms or exosomes released from plasma membranes. The abilities of membrane lipids and proteins/glycoproteins to move between adjacent membrane domains may be related to the extent of their aggregation with similar or different components, the sizes of membrane and cytoplasmic barriers to movements and the complex interactions of these barriers with the cytoskeleton and extracellular matrix [20,21,22,23,39,40,48,49,64]. In the latter case, mucin polymers and long-chain polysaccharides can generate entropic forces that favor or disfavor the projection of cell extensions from the cell surface, as well as control cell shape and immobilize certain cell surface components [65,66].

The actual sizes of membrane domains can vary quite dramatically, depending on domain composition and other factors, from small lipid-only domains or small lipid rafts to rather large, complex glycoprotein–lipid domains that can also have linkages to other structures. Kusumi et al. [20,21,67] have estimated the approximate diameters of various membrane domains, such as micro- and sub-micrometer-sized domains. They are thought to vary in area from 0.04 to 0.24 μm^2^, with approximate transit times of some membrane glycoprotein receptors in such domains ranging from 3 to 30 s. They propose that smaller membrane domains, such as nano- or meso-sized domains in the range of 2–300 nm in diameter, are also present, with complex actin-containing cytoskeletal fence domains in the range of 40–300 nm in diameter compared to entirely lipid raft domains that are usually in the range of 2–20 nm in diameter. Moreover, there are dynamic integral membrane protein complex domains that can vary in size with a minimum range of 3–10 nm in diameter and containing only a few components and a maximum size of at least one-hundred times this diameter [20,21]. The presence of so many different types of cell membrane domains and the selective presence of membrane protein receptors in some of these domains indicate that there is another level of membrane compositional and organizational complexity beyond the original description of the Fluid–Mosaic Membrane Model. This has been called hierarchical membrane organization by Kusumi and his colleagues [20,21].

The proposal that cell membranes are organized into complex hierarchical structures is based on several observations on the variability and dissimilarity of the lateral motions of various cell surface receptors and other membrane components and the ability of cells to quickly reorganize their cell surface membrane structures in order to respond to intracellular and extracellular signals [20,21]. This type of dynamic organization may have evolved so that cells can rapidly respond to ligand binding and other signals. In addition, it may be more efficient to have receptors pre-positioned in the cell membrane within signaling domains so that they can undergo more rapid and specific aggregation into supramolecular transmembrane signaling structures [21]. The presence of barriers or ‘fences’ on the inner plasma membrane surface that limit the lateral motions of specific integral membrane protein components within cytoskeletal-fenced ‘corrals’ or tethering them directly or indirectly to membrane domains may create relatively stable, local membrane domains of high receptor densities. The Kusumi-type hierarchical structures incorporate membrane domains with cell surface receptor diffusion rates 5 to 50 times slower compared to the same components when they are free to diffuse laterally. Over time, such receptors are generally thought to be confined to the specific membrane sub-regions with restricted mobilities [20,21]. This type of organization has been described as important in allowing the pre-positioning of receptors so that they are more fully capable of responding quickly, efficiently and specifically to an appropriate extracellular signal, especially if this type of signal transmission involves the formation of complex signaling platforms [21].

Some fundamental requirements of cell membrane signaling via receptor–ligand binding are thought to consist of a basic Fluid–Mosaic Membrane structure plus various specific membrane nano- and micro-sized domains or compartments capable of forming larger clusters, surrounded by fluid-phase lipids, which can be dynamically trans-membrane linked to cytoskeletal systems [21,22,23,64]. A membrane signaling compartment or signaling domain can be further defined by whether aggregation with similar or different domains occurs as well as their confinement by cytoskeletal fencing or protein ‘fenceposts’ or other properties. Various membrane barriers may be used to prevent large-scale coalescence of smaller membrane domains [20,21].

A variety of different membrane domains and structures probably exist in cell membranes in order to accommodate the large number of possible extracellular signals that cells receive so that particular signals can be distinguished from one another. A facsimile of a Fluid–Mosaic membrane containing lipid raft domains, glycoprotein domains, barrier or ‘corral’ domains and other membrane-associated structures is depicted simplistically in Figure 2 [23]. One should not take such schemes too seriously, because they will likely undergo further changes when new information and data are available. How membrane structures can be affected by domain-clustering agents, such as the binding of various extracellular molecules, changes in ion concentrations and, especially, the integration of lipid molecules from outside the cell in order to change cell and tissue and ultimately host responses, will be the subject of this Special Issue.

In contrast to the very dynamic membrane domains involved in cell signaling, nutrient transport and other properties, cells must also have less dynamic, more stable mosaic membrane structures that are involved in maintaining cell polarity, stable cell–cell interactions and tissue organization. These latter properties of cell membranes require mosaic structures that are not unusually mobile but are more stable, less mobile and integrated and linked to extracellular structures in the pericellular spaces between cells. Such structures could also be (and are often) transmembrane linked to cytoskeletal elements and peripheral membrane networks inside cells to create a fully integrated tissue structural support system [22,23,25,33,34,35,36,65].

## 6. Membrane Vesicles, Globules and Membrane Fusion

Cells are highly dependent on their abilities to capture nutrients and remove cellular waste, such as toxic and damaged molecules. They must transport and transfer various molecules between the extracellular and intracellular microenvironments and between the various organelles and cellular compartments. Cells have to rapidly move various nutrients, structural components and newly synthesized molecules to where they are needed intracellularly and to remove them if they are damaged, degraded or no longer needed. The biosynthesis of molecules inside cells that will eventually be sent to various cell organelles, sub-organelle compartments, the plasma and other membranes, or secreted to the extracellular microenvironment, is generally followed by their immediate binding to specific transport molecules or their packaging into small membrane vesicles that are delivered to specific intracellular membranes or plasma membrane domains at specific membrane contact sites. Alternatively, different intracellular membranes can undergo contact and transient fusion to deliver membrane constituents [68,69,70,71]. Such processes are also used to repair damage to the plasma and intracellular membranes by removing damaged molecules in order to maintain cell function [70,71].

Membrane components, such as membrane lipids, are often moved around and within cells using carriers, such as lipoproteins and lipid-binding proteins, as well as small lipid globules, membrane vesicles and, of course, as mentioned above, intracellular membranes. The carrier membrane vesicles or intracellular membranes eventually fuse with target membranes to deliver their cargo. Periodic membrane–membrane fusion events occur naturally in cells in order to redistribute membrane lipids and other components between different cellular compartments and remove damaged membrane constituents [3,69,70,71,72,73,74]. Membrane fusion ensues during a variety of normal cellular events, such as fertilization, development, endocytosis, secretion, nerve transmission and many other normal developmental and restorative processes. They are also important in many pathologic situations, such as infection, inflammation, neoplasia, cell death and other events [72,73,74].

Inside cells, directed lipid and vesicle transport and their membrane fusion events depend, in large part, on membrane lipid properties, such as the composition, distribution and acylation of lipids in the transport vesicles and in the target membrane domains, as well as the presence of targeting receptor proteins and specific electrolytes at the point of fusion [69,75,76,77,78]. Thus, lipid composition is important in transport vesicles and membranes, as well as in specialized membrane domains that are the targets of fusion events. For example, in some transport systems, sphingolipids are found to be concentrated in vesicles destined to fuse with plasma membranes at the sites of specific membrane domains [76,77,78,79,80]. The presence of specific polyphosphoinositides with their tethered proteins is also important in directed lipid vesicle-mediated transport to the exterior cell surface [76,80,81,82,83]. Once these membrane transport vesicles attach to target membrane domains, membrane fusion follows, which is dependent on the presence of specialized membrane-binding fusion machinery. This fusion machinery requires specific ‘fusogenic’ proteins that pull adjacent membranes together and electrolytes, such as calcium ions, to promote lipid bilayer fusion [71,73,79,80]. The process requires (*i*) the close apposition of the membranes, while counteracting the electrostatic forces between the outer layers of the lipids that will fuse; (*ii*) the destabilization of the bilayer lipid structure, allowing the incorporation of lipids in a non-bilayer transition structure; and (*iii*) the transient reunification into a bilayer lipid structure [76,79].

At the cell membrane inner surface, the presence of membrane fusogenic proteins appears to be essential for directing the fusion process. In plasma membranes, this has been understood to constitute a specialized dynamic membrane microdomain involved in secretion called a ‘porosome’ [76,81]. In some normal cells, porosomes appear as ‘pits’ measuring approximately 0.5–2 μm in diameter with depressions of 100–180 nm in depth [81]. They are involved in directing exocytosis to particular sites at the cell surface. Porosomes are necessary for various normal functions in cells, such as the secretion of proteins, glycoproteins, enzymes, bioregulators and other important molecules [81,82]. Cells also secrete components by releasing or shedding intact plasma membrane microvesicles [83,84,85,86,87,88,89].

As mentioned above, membrane lipid transport occurs inside cells when lipids are attached to carrier proteins or when they are present in micelles, vesicles, globules, membranes and other transport forms [68,69,70,71,77,80,81,82,83,84,85]. These lipid transport systems usually function on a ‘bulk flow’ or ‘mass action’ basis, where the sources that contain higher concentrations of certain membrane lipids can deliver excess lipids to membranes that have lower concentrations of these particular lipids [82]. Once membrane lipids are transported to their destinations, they can also be enzymatically modified in order to reflect the compositions of the membranes at their final destinations [70,80,82]. The bulk flow or mass action delivery of glycerolphospholipids (GPLs) to particular membrane sites may also be used to remove oxidized or damaged lipids from membranes and eventually degrade them or export them from cells [71,81,82].

Cells almost continuously release small 0.1–2 μm diameter membrane vesicles that are derived from budding plasma membranes that extend into the extracellular environment [3,81,83,84]. This is very apparent in cells in tumors, where the transformed cells appear to release many of these vesicles continuously. The released tumor cell vesicles appear as small (<100 nm diameter) microvesicles called exosomes [3,83,84,85,86,87,88,89]. The tumor cell exosomes also contain various non-membrane molecules, such as small DNAs, RNAs, proteins, enzymes, biomodulators, receptors and other molecules [85,86,87,88,89]. The release of small vesicles from normal and tumor cells and their arrival at near and distant cells may constitute a form of cell–cell communication between similar or different cells. This type of membrane exchange of information between cells is not unique. Vesicles released from normal cells have been found in virtually every extracellular fluid, where they appear to play a role in normal cell communication and in the regulation of inflammation, coagulation, development and other normal physiological processes [85,86,87,88]. In tumors, the released membrane vesicles may affect tumor cell–cell interactions as well as tumor–normal cell interactions in the microenvironment, and this has been proposed to promote or at least affect tumor progression, angiogenesis, invasion and metastasis [3,74,86,87,88,89].

Extracellular membrane vesicles, exosomes and other intracellular membrane vesicles can be involved in signaling changes in intracellular levels of calcium, variations in membrane phospholipid content, changes in cellular energy production and many other responses. They can also signal changes in the regulators that can affect cytoskeleton–membrane interactions; membrane-acting enzymes; other effectors of exocytosis, hypoxia and oxidative events; and responses to hydrodynamic stress [85,86,87,88].

## 7. Membrane Lipid Replacement with Dietary Phospholipids

Plasma and intracellular membrane physiology and function depend on the integrity of every category of cellular membrane component, and especially their most venerable components, including unsaturated membrane phospholipids, which are especially susceptible to oxidative damage. When membrane lipids, especially membrane phospholipids, are damaged, degraded or destroyed, they must be repaired or replaced to maintain normal cellular functions and physiology [70,71,82,90,91,92,93,94]. The dietary replacement of damaged membrane phospholipids with undamaged, functional membrane phospholipids is essential for maintaining cellular and tissue functions and for general health [90,91,92,93,94]. To maintain fully functional cellular membranes, animals and especially humans must consume a diet rich in membrane precursors, such as phospholipids and other lipids, proteins, minerals, carbohydrates and other metabolites. This can be difficult to achieve, especially during chronic and acute illnesses; hence, dietary supplements containing membrane phospholipids have been used to supplement and maintain general membrane health [90,91,92,93,94]. Moreover, many of the most sensitive molecules that make up cellular membranes, especially polyunsaturated GPLs, are quite susceptible to oxidative damage and degradation, and membranes unprotected from oxidative and free radical injury have limited half-lives and must be constantly replaced. When orally ingested as foods, membrane phospholipids can be degraded before ingestion or modified within the gastrointestinal system prior to absorption, as well as during transport to cellular destinations, or they can be taken in as intact molecules without degradation or modification. For example, in the gastrointestinal system, excess membrane GPLs can be absorbed as small phospholipid globules and micelles with their constituents basically undegraded, or they can be absorbed in a more specific but less efficient process that utilizes carrier or transfer molecules [68,69,70,71,90,91,92,93,94]. Overall, the process is usually very efficient, and over 90% of ingested phospholipids are normally absorbed and transported into the blood within hours after entering the gastrointestinal system [95]. While in the blood circulation, membrane phospholipids are usually associated with carrier molecules, such as lipoproteins, or the cell membranes of blood cells. However, when they are present in excess amounts compared to fasting levels, they can also be present in small phospholipid globules or micelles. Eventually, they are transported to tissues and cells, where they are transferred by direct membrane contact, endocytosis or by specific carrier and transport proteins into cells. Once inside cells, membrane phospholipids can be moved to various cellular compartments by a number of mechanisms, including membrane–membrane transfer, carrier molecules, small membrane vesicles, lipid globules and chylomicrons, among other mechanisms, to various cellular and organelle membranes [68,77,80,81,82,90,91,92,93].

Dietary membrane phospholipids, such as GPLs from a variety of plant and animal sources, can be enzymatically modified before, during and after their delivery to cells by exchange or modification of their head groups and fatty acid side chains to reflect the specific compositions of their destination membranes. After they have been exchanged or partitioned into their target membrane sites, GPLs and other membrane lipids can be further enzymatically modified to reflect the unique and ever-changing structural and functional membrane needs of various organelles and cells. The entire process of membrane lipid uptake, transport, replacement, exchange and removal is driven overall by a ‘mass action’ or ‘bulk flow’ process [82]. Thus, when particular membrane phospholipids are in great excess during the dietary uptake process, they have an advantage in being able to reach their final cellular destinations with significantly less degradation or free radical oxidation. This ‘mass action’ basis of bulk membrane lipid uptake and transport is also true of the reverse of this process, which eventually results in the exchange and removal of damaged, oxidized phospholipids and their elimination via the gastrointestinal system [69,70,71,80,82].

## 8. Membrane Lipid Replacement in Aging and Chronic Illnesses

Membrane Lipid Replacement (MLR), the use of oral dietary supplements containing essential polyunsaturated GPLs and other membrane lipids, has been successfully used to maintain and recover lost or diminished organelle and membrane function. The most common therapeutic use of oral MLR phospholipids is to treat fatigue and improve mitochondrial function [90,91,92,93,94]. Fatigue is the most common complaint of patients seeking general medical care, and it is associated with aging and most if not all chronic and many acute medical conditions [95,96]. Fatigue is considered a complex, multi-dimensional sensation that is not completely understood at the molecular level, but it is perceived to be associated with a loss of overall energy, extreme mental or physical tiredness, a feeling of exhaustion or diminished endurance or loss of function and an inability to perform even simple tasks without exertion. In aged individuals and in chronic and most acute diseases, fatigue is present due to a variety of causes [90,92,93,96,97]. In patients with moderate to severe fatigue complaints, fatigue has been directly related to the loss of mitochondrial function and diminished production of mitochondrial ATP [98].

In chronic fatiguing illnesses, such as chronic fatigue syndrome and myalgic encephalomyelitis (CFS/ME), long-term fatigue is the most obvious symptom present, or the dominant symptom, and is usually the primary patient complaint [97,98,99,100]. Moreover, in almost all chronic illnesses, fatigue is a common primary or secondary complaint [93,97,98,99,100]. Along with aging, advanced cancers and other diseases, chronic fatigue is a major symptom or complaint. Although severe fatigue is usually related to significant loss of mitochondrial function, mild fatigue is not necessarily related to the loss of mitochondrial function. Mild fatigue can be found in depression and in some psychiatric conditions that are not always related to the loss of mitochondrial function [98,100].

MLR has been used for general health, aging and the treatment of various clinical conditions using oral, antioxidant and environmentally protected GPL [90,91,92,93,94,96]. The oral use of protected GPL has been successfully used for significantly reducing fatigue in patients with chronic fatigue and other chronic illnesses, including CFS/ME, fibromyalgia and other fatiguing illnesses (reviews: [90,91,92,93,96]). For example, in a cross-over clinical study on the effects of oral NTFactor^®^, a mixture of protected GPL, pre- and pro-biotics and other ingredients was used to treat chronic fatigue symptoms in moderately to severely fatigued aged subjects (61–77 years old). It was found that there was good correspondence between the patients’ reductions in fatigue scores (reductions of 35–43%) and their improvements in mitochondrial function tests [101]. This cross-over clinical study indicated that aged individuals with moderate to severe chronic fatigue benefited significantly from taking the MLR oral GPL supplement. They showed significant improvements in fatigue and other clinical parameters during the test arm of the study, but these were slowly reversed when patients were switched to placebo. Their mitochondrial function, as measured by their mitochondrial inner membrane trans-membrane potential, matched the clinical data and showed enhancement up to 45% while on the MLR supplement, but this was significantly reduced after the patients were switched to placebo. When on the MLR supplement, their mitochondrial function tests were similar to results found in much younger subjects (average age of 31 years old), but only if they continued to take the MLR oral supplement [101]. Similar positive results on the effects of the MLR GPL supplement NTFactor^®^ or NTFactor Lipids^®^ on reducing fatigue were found in various patients with chronic fatigue syndrome (CFS/ME), fibromyalgia, Gulf War illness, chronic Lyme disease and various cancers, with reductions in fatigue ranging from 26% to 43% [90,92,93,96,102,103,104,105].

## 9. Membrane Lipid Replacement in Pain Reduction and TRP Channels

MLR supplements, such as NTFactor Lipids^®^, have been used to help reduce a variety of symptoms, including pain, peripheral neuropathy and gastrointestinal symptoms, in chronically ill patients [96,102,105,106]. Pain is a complex phenomenon that can be caused by injury or illness, and it is usually classified according to different criteria based on its pathophysiological mechanism, duration, etiology and anatomical source [107,108]. One type of pain, nociceptive pain, has been described in acute or chronic forms as a sharp or throbbing pain that is often experienced in the joints, muscles, skin, tendons and bones. Nociceptive pain is considered a short-lived condition, although it can also be chronic, generated by the body in response to potentially harmful stimuli, generating reflexes that hypothetically protect the individual from potential damage. This type of pain can be divided into two categories: somatic nociceptive pain, which is usually localized in the dermis, and visceral nociceptive pain, which usually arises as diffuse and poorly defined pain sensations in the midline of the body [108,109]. Multiple events can act on nociceptors to induce pain, and the membrane channels that are involved in nociceptive pain have been identified as transient receptor potential (TRP) channels (TRPV1, TRPM3, TRPA1 etc.) [110]. This large family (>50 subtypes) of membrane channels has been a recent therapeutic target for developing new treatments of chronic pain [111].

In mammals, the TRP channel superfamily consists of 6 subfamilies and 28 members that mainly act as cation channels. TRP channels possess a primary structure that is common to all members, consisting of six trans-membrane domains and one hydrophilic loop that forms the pore that is permeable to monovalent cations and, in certain cases, calcium ions [112]. Certain TRP channels are critical for nociception and thermal sensitivity [98,99]. Previously, it was found that some membrane channels required the presence of GPL phosphoinositides for activity [113,114,115]. TRP channels are regulated by membrane GPL, such as phosphatidylinositol 4,5 bisphosphate or PI(4,5)P2, where the GPL can act as an agonist with desensitization properties, though initially it was described as a general inhibitor of TRP channels. For example, PI(4,5)P2 can inhibit the heat- and capsaicin-activated TRPV1 channels, and the breakdown of this lipid upon phospholipase C activation relieves this inhibition, resulting in the potentiation of TRPV1 activity by pro-inflammatory agents, such as bradykinin [116]. Even if the TRP channels are activated by PI(4,5)P2, they quickly become unresponsive as they become desensitized, losing the ability to be stimulated [117]. Phospholipase C can also catalyze the hydrolysis of PI(4,5)P2, resulting in the formation of the two classical second messengers: inositol 1,4,5 trisphosphate (IP3) and diacylglycerol (DAG). The general view is that the negatively charged head group of PI(4,5)P2 interacts with positively charged residues in the cytoplasmic domains of the TRP channels [118]. This was later confirmed when the co-crystal structures of TRP channels with and without PI(4,5)P2 were published [119]. Though the specific mechanism of action of phosphoinositosides on TRP nociceptor channels is not fully understood (inhibition or activation with desensitization), both proposed mechanisms lead to a final decrease in the activity of these channels either by inhibition or desensitization. Thus, the final result is a decrease in pain sensitivity and nociception promoted by GPL, which is present in MLR supplements, such as NTFactor Lipids^®^ [90,92].

The requirement of higher doses of GPL mixtures to inhibit pain (usually 6 g or more per day of the MLR supplement NTFactor Lipids^®^) may be explained by the fact that PI and its derivatives are not major constituents of MLR supplements [90,92]. Most patients on MLR supplements, such as oral NTFactor Lipids^®^, gradually move to higher daily doses of the MLR supplement to maintain pain control [106]. Currently, we are examining the possible mechanism(s) whereby membrane GPLs alter nerve membrane depolarization and other important properties involved in pain transmission.

## 10. Final Comment

The overwhelming amount of basic information on membrane structure, organization and dynamics, briefly presented here as an overview, has been accepted by the scientific community. However, we are just beginning to understand the role of intracellular membranes and cell membrane properties in explaining various biological phenomena, such as pain. This information will be essential in explaining the complex relationships between cells in tissues and for the eventual development of new therapeutic approaches to overcome various pathological conditions.

## Data Availability

Not applicable.
